# ADC-1013, an agonistic CD40 antibody optimized for local immunotherapy of cancer

**DOI:** 10.1186/2051-1426-1-S1-P42

**Published:** 2013-11-07

**Authors:** Sara M Mangsbo, Sissela Broos, Erika Gustafsson, Christina Furebring, Niina Veitonmäki, Eva Dahlen, Per Norlen, Malin Lindstedt, Thomas Tötterman, Peter Ellmark

**Affiliations:** 1 Alligator Bioscience AB, Lund, Sweden; 2 Immunotechnology, Lund University, Lund, Sweden; 3 Immunology, Genetics and Pathology, Uppsala University, Uppsala, Sweden

## 

Local administration of immune activating antibodies may increase the efficacy and reduce the immune-related adverse events associated with systemic immunotherapy of cancer. Here we report the development of a fully human agonistic CD40 antibody (IgG1), ADC-1013, which has been optimized for local immunotherapy by increasing potency and tumor retention. ADC-1013 activates CD40 receptors on antigen-presenting cells such as dendritic cells, resulting in up-regulation of the co-stimulatory molecules CD80 and CD86, and induction of IL-12. In addition, ADC-1013 induces direct tumor killing of CD40+ tumors, e.g. via antibody-dependent cellular cytotoxicity (ADCC). The anti-tumor effects of ADC-1013 were first assessed in a bladder cancer model (EJ) in immunodeficient NSG mice. Significant anti-tumor responses were demonstrated, and further augmented in mice repopulated with human moDCs/T cells. To study the anti-tumor effects related to the immune activating properties of ADC-1013 in more detail, a human CD40 positive transgenic mouse (hCD40tg) in C57/BL-6 background was used. This transgenic mouse strain has an intact immune system and fully functional dendritic cells that are activated upon ADC-1013 treatment. Furthermore, the dendritic cells obtained from this strain are able to induce antigen specific T cell activation in vitro upon stimulation with ADC-1013. Importantly, treatment with ADC-1013 in a syngeneic bladder cancer (MB49) model, which is hCD40 negative, demonstrated that ADC-1013 induce significant tumor protection and long term immunity independent of direct tumor targeting. In addition, the anti-tumor immunity was shown to be T-cell dependent. To our knowledge, ADC-1013 represents the first immunomodulatory antibody optimized for local immunotherapy of cancer. It is currently in late pre-clinical development and will enter clinical trials in 2014.

